# Competing endogenous RNA networks in human cancer: hypothesis, validation, and perspectives

**DOI:** 10.18632/oncotarget.7266

**Published:** 2016-02-08

**Authors:** Chao Yang, Di Wu, Lin Gao, Xi Liu, Yinji Jin, Dong Wang, Tianzhen Wang, Xiaobo Li

**Affiliations:** ^1^ Department of Pathology, Harbin Medical University, Harbin, China; ^2^ Department of Obstetrics and Gynecology, First Affiliated Hospital of Harbin Medical University, Harbin, China; ^3^ Center for Endemic Disease Control, Chinese Center for Disease Control and Prevention, Harbin Medical University, Harbin, China; ^4^ Department of Cardiovascular Disease, Inner Mongolia People's Hospital, Hohhot, China; ^5^ College of Bioinformatics Science and Technology, Harbin Medical University, Harbin, China

**Keywords:** competing endogenous RNA, cancer, miRNAs, lncRNA, pseudogene

## Abstract

Non-coding RNAs represent a majority of the human transcriptome. However, less is known about the functions and regulatory mechanisms of most non-coding species. Moreover, little is known about the potential non-coding functions of coding RNAs. The competing endogenous RNAs (ceRNAs) hypothesis is proposed recently. This hypothesis describes potential communication networks among all transcript RNA species mediated by miRNAs and miRNA-recognizing elements (MREs) within RNA transcripts. Here we review the evolution of the ceRNA hypothesis, summarize the validation experiments and discusses the significance and perspectives of this hypothesis in human cancer.

## THE EMERGING OF THE CERNA HYPOTHESIS

MicroRNAs (miRNAs) are a class of small non-coding RNAs of∼22 nucleotides in length. MiRNAs majorly degrade mRNAs or inhibit translation of mRNAs by binding to miRNA response elements (MREs) on target RNA transcripts [1]. Occasionally, miRNAs can enhance the gene expression or increase the translation of their targets in some conditions [2]. For years, it is believed that miRNAs regulate gene expression in a simple “miRNA→mRNA→protein” pattern. However, some observations are unable to be explained with this regulation pattern [3]. Firstly, miRNA target prediction algorithms suggest that each miRNA has tens to hundreds of targets [4, 5], and high throughput techniques used for identifying the interactions of endogenous miRNA-targets (such as PAR-CLIP and HITS-CLIP) show large number of miRNA binding sites in their target mRNA sequences [6, 7]. However, this observation is not consistent with the fact that many phenotypic changes caused by miRNA mutants can be rescued by the mutation of a single target [8, 9]. Secondly, bioinformatics research suggests that 15-30% of mammary genes can be regulated by miRNAs [10]. However, upon knockdown of the Dicer-1 gene and subsequent abolishment of miRNA maturation, the expression of only about 4% of transcribed genes increase [11]. Finally, miRNA-mediated protein changes are typically only within the two-fold range [4, 5]. However, even larger changes in the expression of some proteins will be tolerated by cells and not lead to any obvious changes in phenotype [12]. Taken together, these observations imply that many endogenous miRNA-target interactions may have no functional role. So then what is the significance of the presence of miRNAs and large miRNA binding sites across the transcriptome?

In 2007, Javier et al. reported that non-coding IPS1RNA altered the level of PHO2 protein in plants by sequestering the availability of miR-399 and preventing it from inhibiting the stability and translation of PHO2 mRNA [13]. Several weeks later, Ebert et al. introduced a method of miRNA sequestration in animals called the “miRNA sponge”, reducing miRNA availability for its mRNA targets [14]. These early studies show that miRNA activity could be regulated by “target mimics,” and suggest that miRNA-target interactions may be bilateral instead of unilateral. In 2009, based on the findings of the previous studies, Seitz proposed that many natural miRNA target sites could act as miRNA decoys, and changes in the expression of some miRNA targets would alter the availability of an miRNA and thus affect the activity of its other targets [3].

**Figure 1:**
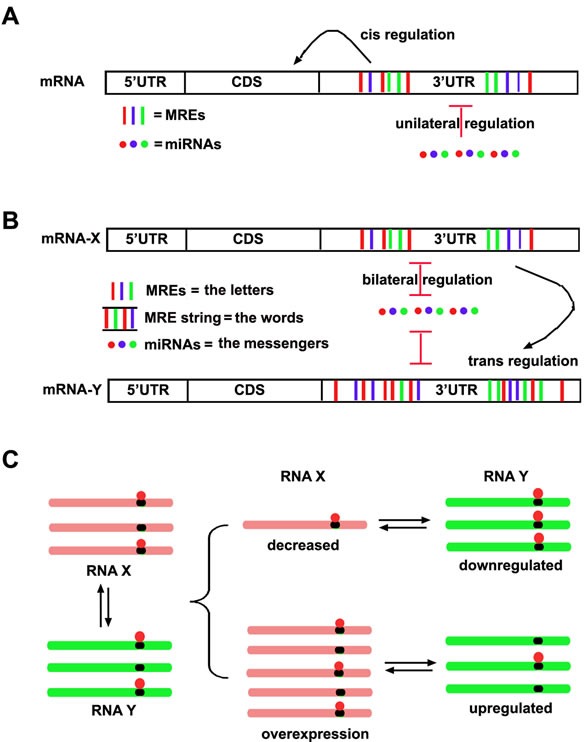
The schematic ceRNA hypothesis **A.** The conventional model about interaction between miRNAs and mRNAs. It has been believed that miRNA is an “initiator” and unilaterally regulates mRNA expression, while the MREs within mRNAs in cis regulate the stability and translation of mRNA itself. **B.** The ceRNA model about the interaction between miRNAs and mRNAs. Not only miRNAs can regulate mRNAs, but also miRNAs can be regulated by mRNAs reversely. Thus MREs within an mRNA can in cis regulate itself and in trans regulate other mRNAs. Different RNA transcripts communicate through a specific “RNA language”. MREs are the “letters” of the language, while the “words” consisting of MRE string within RNA transcript would be recognized and conveyed to other RNA transcripts by the miRNA “messenger”. **C**. The regulation pattern of ceRNAs. Multiple RNA transcripts containing MREs to compete same miRNA pool, thus alteration of any RNA transcripts would result in the same directional change of other RNA transcripts targeted by same miRNA pool.

In 2010, Pandolfi et al. found that some protein-coding genes and their pseudogenes contain the same conservative miRNA binding sites in their 3′UTRs, and that they can regulate their respective expression levels by competing for miRNA binding [15]. Based on these findings, one year later, Pandolfi et al. proposed the competing endogenous RNA (ceRNA) hypothesis [16]. In this hypothesis, MREs are viewed as the letters of an “RNA language” and the transcript RNAs with specific MREs can communicate with others via the “miRNA messenger.” Any RNA transcript possessing MREs may function as a ceRNA and de-repress the activity of other RNAs with similar MREs by competing for the same miRNAs in the available miRNA pools (Figure 1).

According to the ceRNA hypothesis, the role of miRNAs in regulating gene expression has thus been amended from that of an “initiator” to a “mediator,” and the regulation pattern has been amended from liner (miRNA→mRNAs) to network-based (RNAs→miRNAs→mRNAs). Additionally, the ceRNA hypothesis provides a reasonable justification for the presence of pseudogenes, long non-coding RNA (lncRNA) and circular RNA (circRNA).

## COMPONENTS OF ceRNA NETWORKS

Two protagonists are necessary for ceRNA networks: miRNAs as messengers and transcripts as ceRNA. Each miRNA has numerous RNA targets. A single miRNA binding to MREs in one target transcript would relieve the repressive activity of that miRNA on other target genes; thus any transcript harboring one or more MREs has the potential to be a ceRNA of the miRNA target transcripts, including mRNA, pseudogenes, circRNAs, and lncRNAs.

The human transcriptome comprises about 20,000 protein-coding mRNA transcripts [17] with many of those having multiple MREs in their 3′UTR [18]. Previous studies have indicated that miRNA can decrease the stability of mRNAs or inhibit their translation by binding to MREs [19]. Pseudogenes are DNA fragments whose sequences are similar to known functional genes but have no protein coding functions due to accumulated mutations during the evolutionary process [20]. About 11,000 pseudogenes have been found in the human genome [21]. Notably, transcribed pseudogenes possess many of the same MREs in their 3′UTRsas their protein-coding counterparts [22]. LncRNAs are non-coding RNAs of greater than200 nucleotides in length. To date, 10,000-32,000 lncRNA transcripts have been identified using high throughput sequencing techniques [23]. LncRNAs were recently found to contain MREs as well as function as miRNA sponges [24]. CircRNAs are non-coding RNAs that form a covalent closed loop by the direct ligation of 5′ and 3′ ends of linear RNAs [25], which are stable and present at levels comparable to their canonical counterparts [26]. Recent studies suggest that circRNAs are among the significant components of ceRNA networks also [27-29].

In addition to mRNAs, pseudogene transcripts, lncRNAs, and circRNAs, the human transcriptome also contains many hundreds of thousands of small RNAs, including transfer RNAs involved in translation of mRNAs, small nuclear RNAs involved in splicing, small nucleolar RNAs involved in ribosomal RNA modification, PIWI-interacting RNAs involved in transposon repression, and transcription initiation RNAs involved in transcription regulation [30]. Importantly, at less than 200 nucleotides in length, these small RNAs may not contain enough MREs to compete with the larger RNA species; however, they may be involved in the regulation of ceRNA networks via regulating abundance and availability of protagonists at transcriptional and post-transcriptional levels.

## VALIDATED ceRNAS IN HUMAN CANCER

Linc RNAs, mRNAs, pseudogene transcripts and circRNAs are all revealed to be involved in the tumorigenesis and progression of human malignant tumors via the ceRNA mechanism (Table 1).

**Table 1:** A summary of validated ceRNAs in human cancer

ceRNA species		Corresponding ceRNAs	Shared miRNAs	Cancer type	Involved functions	Ref
mRNA	PTEN	ZEB2	has-miR-181-5p, hsa-miR-200b-3p, hsa-miR-25-3p, hsa-miR-92a-3p	Melanoma	Proliferation	[32]
		13 genes^a^	It is predicted that PTEN and RB1 share MRE for 32 common miRNAs	Glioblastoma	Cell growth	[35]
		VAPA, CNOT6L	hsa-miR-17-5p, hsa-miR-19a-3p, hsa-miR-20a-5p, hsa-miR-20b-5p, hsa-miR-26b-5p, hsa-miR-106a-5p, hsa-miR-106b-5p, hsa-miR-19b-3p	Prostate cancer	Proliferation	[36]
	Versican 3′-UTR	RB1, PTEN	hsa-miR-144-3p, hsa-miR-136-5p, hsa-miR-199a-3p	Breast carcinoma	Proliferation	[37]
		Versican, CD34, Fibronectin	hsa-miR-199a-5p, hsa-miR-144-3p, hsa-miR-431-5p	Hepatocellular carcinoma	Proliferation, apoptosis, migration, invasion	[38]
	CD44 3′-UTR	CD44 a, CDC42	hsa-miR-216a-5p, hsa-miR-330-3p, hsa-miR-608	Breast cancer	Proliferation, apoptosis, angiogenesis	[39]
		CD44, Col1α1, FN1	hsa-miR-328-5p, hsa-miR-512-3p, hsa-miR-491-5p, hsa-miR-671-5p	Breast cancer	Migration, invasion, adhesion	[40]
	FOXO1 3′UTR	E-cadherin	hsa-miR-9-5p	Breast cancer	Epithelial-to-mesenchymal transition, metastasis	[91]
	AEG-1	Snail, Vimentin	hsa-miR-30a-5p	Non-small cell lung cancer	Epithelial-to-mesenchymal transition	[92]
	Hmga2	Tgfbr3	has-let-7s	Lung cancer	Transformation, progression	[41]
	OCT4B	OCT4A	hsa-miR-145-5p, hsa-miR-20a/b-5p, hsa-miR-106a/b-5p, hsa-miR-335-5p	Cancer cell lines^b^	Proliferation	[45]
Pseudogenes	PTENP1	PTEN	hsa-miR-17-5p, hsa-miR-21-5p, hsa-miR-214-3p, hsa-miR-19-3p, hsa-miR-26a-5p	Prostate cancer	Proliferation	[15]
		PTEN	hsa-miR-21-5p	Renal cell carcinoma	Proliferation, invasion, chemosensitivity	[22]
		PTEN	hsa-miR-17-5p, hsa-miR-19b-3p, hsa-miR-20a-5p	Hepatocellular carcinoma	Proliferation, migration/invasion, autophagy, apoptosis	[46]
		PTEN	Unknown	Gastric Cancer	Proliferation, apoptosis, migration, invasion	[47]
	KRAS1P	KRAS	hsa-miR-143-3p, hsa-let-7s	Prostate cancer	Cell growth	[15]
	BRAFP1	BRAF	hsa-miR-134-5p, hsa-miR-543, hsa-miR-653-5p	Lymphoma	Proliferation	[49]
	CYP4Z1	CYP4Z2P	hsa-miR-211-5p, hsa-miR-125a-3p, hsa-miR-197-3p, -hsa-miR-1226-3p, hsa-miR-204-5p	Breast cancer	Angiogenesis	[53]
	HMGA1P6/7	HMGA1	miRNAs targeting the HMGA1	Pituitary tumors	Proliferation, migration	[56]
	OCT4-pg4	OCT4	hsa-miR-145-5p	Hepatocellular carcinoma	Cell growth	[93]
LncRNAs	HULC	PRKACB	hsa-miR-372-5p	Hepatocellular carcinoma	Chromatin accessibility	[60]
	PTCSC3	unknown	hsa-miR-574-5p	Thyroid cancers	Cell growth, cell cycle, apoptosis	[62]
	Linc-RoR	Oct4, Sox2, Nanog	hsa-miR-145-5p	Endometrial cancer stem cells	Differentiation	[64]
	HOTAIR	HER2	hsa-miR-331-3p	Gastric Cancer	Proliferation, migration and invasion	[66]
	Linc00974	KRT19	hsa-mir-642a	Hepatocellular carcinoma	Proliferation, invasion	[68]
	H19	Vimentin, ZEB1, ZEB2	hsa-miR-138-5p, hsa-miR-200a-3p	Colorectal cancer	Epithelial to mesenchymal transition	[70]
	HOST2	HMGA2,c-Myc, Dicer, Imp3	hsa-let-7b-5p	Ovarian cancer	Migration, invasion and proliferation	[71]
circRNAs	cir-ITCH	ITCH	hsa-miR-7-5p, hsa-miR-17-5p, hsa-miR-214-3p	Esophageal squamous cell carcinoma	Cell growth	[72]
		ITCH	hsa-miR-7-5p, hsa-miR-20a-5p, hsa-miR-214-3p	Colorectal Cancer	Cell growth	[73]

a ABHD13, CCDC6, CTBP2, DCLK1, DKK1, HIAT1, HIF1A, KLF6, LRCH1, NRAS, RB1, TAF5, TNKS2

b ovarian teratoma cell line, gastric cancer cell lines, prostate cancer cell line, colon cancer cell line

### mRNAs as ceRNAs in human cancer

PTEN is a key tumor suppressor gene that encodes a phosphatase that antagonizes the oncogenic PI3K/Akt signaling pathway by converting phosphatidylinositol 3,4,5-trisphosphate to phosphatidylinositol 4,5-bisphosphate [31]. In 2011, Pandolfi et al. were the first to identify several PTEN ceRNAs, including ZEB2 [32], and found that their loss promotes tumorigenesis in a mouse model of melanoma. Many studies have indicated that elevated ZEB2 expression is associated with poor prognosis in multiple tumors through acting as the epithelial-to-mesenchymal transition (EMT) regulator [33, 34]. The Pandolfi study shows that ZEB2 mRNA is a PTEN ceRNA that enhances PTEN expression in a 3′UTR-dependent, miRNA-dependent, and protein coding-independent manner. Loss of ZEB2 transcript can activate the PI3K/Akt pathway by decreasing PTEN, thus promoting cell transformation. This study highlights the ability of an mRNA and its respective protein to potentially exert different biological functions. Califano et al. also validate 13 miRNA-mediated PTEN regulators, showing that their deletions caused decrease of PTEN in a 3′UTR-dependent manner, subsequently promoting tumor cell growth [35]. Both studies are the first to report on protein-coding mRNAs acting as non-coding RNAs. Pandolfi et al. recently employ computational analysis and experimental validation to prove the presence of the PTEN ceRNA network in prostate cancer cells [36]. This network includes many protein-coding mRNAs, such as SERINC1, VAPA, and CNOT6L, which share MREs with the PTEN transcript and can therefore act as ceRNAs, regulating the level of PTEN transcript by competing for the same miRNAs.

Versican is a chondroitin sulphate proteoglycan present in the extracellular matrix. Yang et al. find that the versican 3′UTR can bind to and modulate miRNA activity, and subsequently increase the translation of tumor suppressors RB1 and PTEN in breast carcinoma cells [37]. In 2013, this group reported that the versican 3′UTR induces the development of hepatocellular carcinoma (HCC) and demonstrated that the versican 3′UTR can increase the expression of versican, CD34, and fibronectin via a ceRNA mechanism in HCC cells [38]. Similarly, this group also validated the 3′UTR of CD44 as a ceRNA for several transcripts in breast carcinoma cell lines [39, 40]. In one study, they showed that the CD44 3′UTR serves as a competitor for hsa-miR-216a-5p, hsa-miR-330-3p, and and hsa-miR-608 to increase CD44 and CDC42 protein levels in the breast cancer cell line MT-1, resulting in inhibition of cell proliferation and tumor-formation, promotion of angiogenesis, and induction of apoptosis [39]. In another study, they indicated that the CD44 3′UTR is also the ceRNA for collagen type 1α1 (Col1α1) mediated by hsa-miR-328-5p and the ceRNA for fibronectin type 1 (FN1) mediated by hsa-miR-512-3p, hsa-miR-491-5p, and hsa-miR-671-5p [40]. Exogenous CD44 3′UTR increased the expression of CD44, Col1α1, and FN1, resulting in enhanced cell motility, invasion, and cell adhesion in the breast cancer cell line MDA-MB-231. These results suggest that certain mRNA transcripts may have distinct roles in different cancer types or even among different cell lines of the same cancer type.

A recent study indicated that Hmga2 mRNA can promote lung cancer progression through ceRNA function independent of its protein-coding function [41]. Hmga2 mRNA has seven conserved has-let-7s binding sites in its 3′UTR [42]. Overexpression of Hmga2 titrates away has-let-7s from Tgfbr3, freeing and increasing Tgfbr3 to triggerAgo2 and TGF-β signaling pathway activity, eventually leading to lung cancer progression.

Octamer-binding transcription factor 4 (OCT4) encodes a transcription factor containing a POU homeodomain that plays a key role in embryonic development and stem cell pluripotency [43]. Aberrant expression of this gene is associated with tumorigenesis [44]. The OCT4 gene has three mRNA isoforms: OCT4A, OCT4B, and OCT4B1. Zheng et al. reported that OCT4B functioned as a ceRNA to regulate OCT4A expression in an miRNA-dependent manner in several tumor cell lines [45]. This is the first report showing spliced gene isoforms serving as ceRNAs.

### Transcribed pseudogenes as ceRNAs in human cancer

Pandolfi et al. found that both PTEN and PTENP1 are both reduced in prostate cancer and that overexpression of PTENP1 increase the expression of PTEN and suppress tumor growth. Further they revealed that PTENP1 acts as a decoy for PTEN-targeting miRNAs to de-repress PTEN expression [15]. This study verifies for the first time that pseudogenes are not only evolutionary artifacts, but rather regulators contributing to post-transcriptional regulation of their parental genes. Later studies of clear-cell renal cell carcinoma, HCC, and gastric cancer identify this same mode of PTENP1 ceRNA regulation [22, 46, 47]. In the same report, Pandolfi et al. also examined other cancer-related pseudogenes and genes, and found miRNA binding sites to be well conserved. Of them, KRAS and its pseudogene KRAPS1P are found to be positively correlated in prostate cancer. Overexpression of the KRAPS1P 3′UTR increase KRAS mRNA abundance and promote tumor cell growth [15]. Recently, Morris et al. also characterized a previously unidentified antisense PTENP1 (asRNA) as a ceRNA [48]. The PTENP1 antisense beta isoform can interact with PTENP1 through an RNA-RNA binding model and decrease PTENP1 stability and miRNA sponge activity. In 2015, Pandolfi's group provided additional evidence to support the role of pseudogenes participating in cancer development as ceRNAs [49]. The BRAF gene encodes a protein belonging to the raf/mil family of serine/threonine protein kinases [50]. This protein regulates the MAP kinase/ERK signaling pathway and affects cell division and differentiation [51]. Pandolfi et al. showed that the BRAF pseudogene, BRAFP1, was often aberrantly expressed in multiple human cancers, and overexpression of the murine BRAF pseudogene induced lymphoma formation in mice. It was found that the BRAF pseudogene acted as a ceRNA to elevate BRAF expression and activate MAPK, confirming the oncogenic potential of BRAFP1 [49].

CYP4Z1 encodes a member of the cytochrome P450 superfamily of enzymes, and the cytochrome P450 proteins are monooxygenases which catalyze many reactions in organisms [52]. CYP4Z1 and its pseudogene, CYP4Z2P, share many MERs in their 3′UTRs. Overexpressed CYP4Z2P can function as ceRNAs to increase CYP4Z1 expression, resulting in increased tumor angiogenesis via phosphorylation of ERK1/2 and PI3K/Akt in breast cancer [53].

It has been reported that Hmga1 overexpression induces the formation of pituitary tumors in vivo [54]. However, Hmga1 overexpression is not related to any rearrangement or amplification of the Hmga1 locus in these tumors [55]. Fusco et al. found that Hmga1 pseudogene expression was significantly correlated with Hmga1 transcript levels. Acting as ceRNAs, these pseudogenes elevate the expression of Hmga1 and other cancer related genes, enhancing the proliferation and migration of pituitary tumor cells [56].

Recently, Prins et al. applied a novel bioinformatic methodology for measuring pseudogene transcription from RNA-seq data in 819 breast cancer samples [57]. They found 440 pseudogenes with high confidence transcribed in breast cancer tissues, of which 309 exhibited significant differential expression among breast cancer subtypes. Furthermore, 177 transcribed pseudogenes are predicted to function as ceRNA of their parent genes. This study thus suggests that transcription of pseudogenes, acting as ceRNAs, may play a larger and more extensive role in cancer.

### LncRNAs as ceRNAs in human cancer

HULC is the first long non-coding RNA identified to be specifically and highly increased in HCC [58]. It is not only a potential novel biomarker for HCC but also a regulator for tumor cell proliferation [59]. In 2010, Sun et al. explained the regulation network of HULC as a ceRNA in HCC [60]. They showed that HULC functions as a miRNA sponge to inhibit the activity of many miRNAs, including hsa-miR-372-5p, resulting in de-repression of its target gene, PRKACB. PRKACB, acting as the catalytic subunit of PKA, induces phosphorylation of cAMP response element binding protein (CREB), which in turn enhances CREB-dependent HULC increasing in HCC. This study thus provides the first evidence of lncRNAs acting as ceRNAs.

Papillary thyroid carcinoma susceptibility candidate 3 (PTCSC3) is a newly identified and highly thyroid-specific non-coding RNA [61]. PTCSC3 is dramatically decreased in thyroid cancers and has the characteristics of a tumor suppressor. Transfection of PTCSC3 into thyroid cancer cells results in significant growth inhibition, cell cycle arrest, and increased apoptosis. These effects are related to the ability of PTCSC3 to bind hsa-miR-574-5p as ceRNA [62].

The large intergenic non-coding RNAlinc-RoR is first identified inhuman embryonic stem cells (ESCs). As a ceRNA, linc-ROR shares MREs with core transcription factors, such asOct4, Nanog, and Sox2, and prevents these transfection factors from miRNA-mediated suppression in self-renewal of ESCs [63]. Linc-RoR can also increase transcription factor levels by sequestering hsa-miR-145-5p, thereby inhibiting cancer stem cell differentiation [64]. These results suggest that lncRNA ceRNAs participate in both embryonic development and carcinogenesis.

HOTAIR is an lncRNA involved in the development of multiple cancers by interacting with polycomb repressive complex 2 (PRC2) [65]. Wang et al. provides evidence that HOTAIR may also function as a ceRNA by increasing the expression of human epithelial growth factor receptor 2 (HER2) through competition for hsa-miR-331-3p in the pathogenesis of gastric cancer [66].

Linc00974 is an lncRNA located upstream of the protein-coding gene KRT19, which is recently characterized as an HCC progression-associated factor [67]. A recent study suggests that overexpression of linc00974 can interact with hsa-miR-642a-5p as a ceRNA, leading to the increase of KRT19 and subsequent activation of Notch and TGF-β signaling pathways, to increase proliferation and invasion of HCC [68].

LncRNA H19 is expressed exclusively from the maternal allele and is involved in the growth and development of multiple cancer types [69]. Recent data show that H19 triggers EMT progression in colorectal cancer by binding hsa-miR-138-5p and hsa-miR-200a-3p, antagonizing their functions and leading to the increase of their endogenous targets Vimentin, ZEB1, and ZEB2 [70].

HOST2, a human ovarian cancer-specific lncRNA, is first identified in 2003, but its function and mechanism in ovarian cancer progression is not explained until 2015 [71]. It is now known that HOST2 promotes tumor cell migration, invasion, and proliferation in epithelial ovarian cancer by functioning as a sponge of has-let-7s, a potent tumor suppressor.

Taken together, these studies highlight the function of lncRNAs as ceRNAs in cancer.

### CircRNAs as ceRNAs in human cancer

CircRNAs are a more recently identified RNA transcript, so their ceRNA activity has not yet been as thoroughly investigated as that of other transcripts. Cir-ITCH is a circular RNA that spans several exons of ubiquitin (Ub) protein ligase (E3) (ITCH). Recently, two groups report that cir-ITCH functions as a ceRNA in human cancer [72,73]. Zhou et al. reports that cir-ITCH expression is generally lower in esophageal squamous cell carcinoma compared to surrounding peritumoral tissue. Moreover, overexpression of cir-ITCH might increase ITCH levels by acting as a sponge of hsa-miR-7-5p, hsa-miR-17-5p, and hsa-miR-214-3p, allowing ITCH to promote ubiquitination and degradation of phosphorylated Dvl2, then inhibiting the Wnt/β-catenin pathway [72]. Similar results are found in another study by Chen et al focusing on colorectal cancer [73].

## PERSPECTIVES OF CERNAS IN HUMAN CANCER

Since its proposition in 2011, the ceRNA hypothesis has been validated by many studies. The ceRNA hypothesis provides a new way to view and understand complicated RNA networks. For example, the distinction between a coding and a non-coding RNA is now somewhat ambiguous. The finding of the mRNA of oncogene ZEB2 suppressing melanoma development suggests that a traditional protein-coding mRNA may not only function as a non-coding RNA, but also the role of the protein may be different or in opposition to that of the non-coding RNA. Moreover, according to the ceRNA model, it is reasonable to deduce that a certain transcript may have different roles in different tumor types, depending on the availability of the miRNA pool and other transcripts of the ceRNA regulatory network. For instance, the versican 3′UTR has been identified to play different roles in breast cancer and HCC [35, 36]. Thus, identifying the different or even opposing roles of a single mRNA's coding and non-coding character at different settings would strengthen the ceRNA hypothesis.

The potential of RNAs for use as diagnostic and prognostic biomarkers has received a lot of attention in recent years. For example, RNA transcripts HOST2 and Hsa_circ_002059 have been identified as biomarkers for ovarian cancer and gastric cancer, respectively [74, 75]. The ceRNA model not only suggests the existence of a complex regulatory network in cancer, but also implies the possibility of using a panel of network molecules to diagnosis and predict cancer [76]. Additionally, the ceRNA model partially explains the relationship between single nucleotide polymorphisms (SNPs) and cancer prediction, as SNPs locate in miRNA binding sites might regulate miRNA binding. Recently, 30 proto-oncogene-associated SNPs were reported to interrupt the interaction between miRNAs and their targets, and some of these SNPs can predict the therapeutic outcome of cancer patients [77].

The ceRNA model also provides an opportunity to discover the novel therapeutic targets for human cancer. As mentioned above, mRNAs of Hmga2 and BRAF could induce lung cancer progression and lymphoma formation respectively, which suggests these mRNAs may be a therapeutic targets for these malignant diseases. In addition, artificial miRNA sponges, which contain multiple miRNA binding sites and thus imitate the characteristics of ceRNAs as miRNA inhibitors, bring a new platform for RNA-based therapeutic applications in clinic [14, 78].

There are still some challenges to hamper the application of the ceRNA model to diagnose, predict or treat human cancer. For instance, what cellular conditions must exist for the ceRNA network to exist? It seems that the relative concentration of ceRNAs and their target miRNAs must be suitable for competition. Not only the absolute expression, but also the numbers and effectiveness of MREs to bind miRNAs, the subcellular localization, interaction with RNA binding proteins, and even RNA editing are factors that have an important influence on RNA transcripts to act as ceRNAs [30]. Recently, Markus et al. challenged the feasibility of the ceRNAhypothesis [79]. They find that hsa-miR-211-5p target genes only increased when over-expressed AldoA mRNA (a validated target of hsa-miR-122-5p) approach a threshold of 1.5× 10^5^ transcripts per cell, which exceeds the physiological levels of any endogenous target [79]. However, the method of assessing endogenous hsa-miR-122-5p target changes in their study is real-time PCR, which is not commonly used to measure alterations of miRNA targets because miRNAs usually inhibit the translation of their targets without leading to their degradation [80]. Additionally, hsa-miR-122-5p levels in the HCC cells reach 1.2×10^5^ transcripts per cell, which is among the highest reported for a miRNA in any mammalian system. Thus, this is an extreme example used for analysis, and the results may not be reproduced with other moderately abundant miRNAs.

Theoretically, ceRNA networks consist of any RNA transcripts and the miRNAs that recognize and bind the MREs within those RNA transcripts. Thus, identification of MREs within RNA transcripts is essential for further study of ceRNA networks. Target Scan, Pic Tar and some other database represent useful tools to identify putative miRNAs and mRNAs crosstalk. However, these database only consider the binding of miRNAs within the 3′ UTR of mRNAs, while recent researches suggest that ∼40% of the miRNA binding sites are located within coding regions of mRNAs [6, 7], thus nearly 50% of information are missing by using these database to analysis the crosstalk between miRNAs and mRNAs. Additionally, there are no effective and efficient tools available to predict the interaction between miRNAs and other RNA transcripts until now. Thus bioinformatics techniques should therefore are urgent to extract ceRNA networks contributing to cancer progression from large databases. Recently, some bioinformatics analysis strategies to identify ceRNA networks in cancer are emerging. For insistence, the ceRDB database is established to predict mRNAs that may act as ceRNAs of other mRNAs by examining the co-occurrence of MREs on the 3′UTRs of genome-wide mRNA transcripts, with data pulled from Target Scan release 5.2 [81]. Paci et al. propose a novel computational approach suitable for exploring the potential role of lncRNAs as ceRNAs. Using this approach, they find multiple lncRNAs functioning as ceRNAs in normal and cancerous breast tissues [82]. Guo et al. analyzes lncRNA microarray data in gastric cancer and use bioinformatics technology to construct an lncRNA-miRNA-mRNA network. This study shows a clear caner-associated ceRNA regulatory network, including 9 lncRNAs, 13 miRNAs and multiple mRNAs involved in cell proliferation, apoptosis, invasion, and metastasis [83]. In addition, Suman et al. compiles a database of human disease-circRNA associations using bioinformatics [84, 85]. Our group has also established several cell death databases based on miRNAs mediated networks [86-90].

In conclusion, even as the ceRNA hypothesis is still in its infancy, its ability to contribute to the understanding of tumorigenesis, cancer progression, and cancer therapy development is continually being validated.
